# The changing landscape of gene expression analysis

**DOI:** 10.1093/bib/bbag185

**Published:** 2026-04-24

**Authors:** Qiongyi Zhao, Sophie Shen, Woo Jun Shim, Nathan J Palpant

**Affiliations:** Institute for Molecular Bioscience, The University of Queensland, 306 Carmody Road, St Lucia, Brisbane QLD 4072, Australia; Institute for Molecular Bioscience, The University of Queensland, 306 Carmody Road, St Lucia, Brisbane QLD 4072, Australia; Institute for Molecular Bioscience, The University of Queensland, 306 Carmody Road, St Lucia, Brisbane QLD 4072, Australia; Institute for Molecular Bioscience, The University of Queensland, 306 Carmody Road, St Lucia, Brisbane QLD 4072, Australia

**Keywords:** gene expression analysis, 25-year trajectory, bibliometrics, transcriptomic knowledgebases, foundation models for gene expression analysis, programmable regulatory design

## Abstract

Gene expression analysis has evolved substantially over the past 25 years, from early transcript surveys using expressed sequence tags and microarrays to RNA sequencing, and more recently to single-cell and spatial transcriptomics. These successive waves have expanded measurement scale and resolution, enabling systematic discovery of transcriptional programmes, inference of gene regulatory networks, and increasingly direct links between transcriptomic insight and therapeutic strategies that modulate gene expression. In this Perspective, we synthesize major methodological milestones with bibliometric trends in leading bioinformatics journals to describe four revolutions that redefined gene expression analysis. We also map widely used computational tools onto a common timeline by analysing 70 78 831 open-access full-text articles, illustrating how enduring statistical frameworks coexist with rapidly growing end-to-end analysis ecosystems. We highlight current challenges and emerging directions in core bioinformatics approaches for gene expression analysis. Looking ahead, we argue that the next era will be defined less by generating new datasets and more by organizing, searching, and reusing transcriptomic and multimodal information at scale. We propose three future directions: consortium-scale searchable transcriptomic knowledgebases, foundation models for gene expression analysis, and programmable regulatory design for engineered control of gene expression. The landscape of gene expression analysis is shifting from descriptive measurement towards queryable, predictive, and programmable gene expression biology.

Key Points

- This Perspective traces four major revolutions in gene expression analysis over the past 25 years: expressed sequence tags and microarrays, RNA sequencing, single-cell transcriptomics, and spatial and multimodal profiling, and discusses how they reshaped experimental practice and concepts in the field.
- Bibliometric trends in leading bioinformatics journals mirror these shifts; analysis of 70 78 831 PubMed Central open-access full-text articles highlights how long-standing statistical frameworks coexist with rapidly growing end-to-end analysis ecosystems.
- We highlight current challenges in core bioinformatics approaches for gene expression analysis, including usability, maintenance, reproducibility, and parameter transferability across datasets.
- We argue that the next era will be defined less by generating new datasets and more by organizing, searching, and reusing transcriptomic and multimodal information at scale.
- We propose three future directions: consortium-scale searchable transcriptomic knowledgebases, foundation models for gene expression analysis, and programmable regulatory design for engineered control of gene expression.

## Introduction

Few analytical frameworks have transformed the study of biology as profoundly as gene expression analysis. From the earliest scalable transcript surveys in the 1990s [1–3] to the recent explosion of single-cell [4, 5] and spatial [6, 7] technologies, gene expression analysis has shaped how we understand cellular identity, developmental processes, and disease mechanisms. Initially focused on bulk measurements of average gene abundance, gene expression analysis has now become a central pillar of systems biology, enabling researchers to map cellular heterogeneity [8, 9], infer gene regulatory networks [10, 11], and predict patterns of gene expression or regulatory activity directly from genomic sequences [12, 13]. Importantly, the ability to map what genes are expressed where has helped translate transcriptomic insights into therapeutic strategies that modulate gene expression, spanning gene and RNA medicines such as RNA interference (siRNA therapeutics) and antisense oligonucleotides (ASOs) [14, 15].

**Figure 1:**
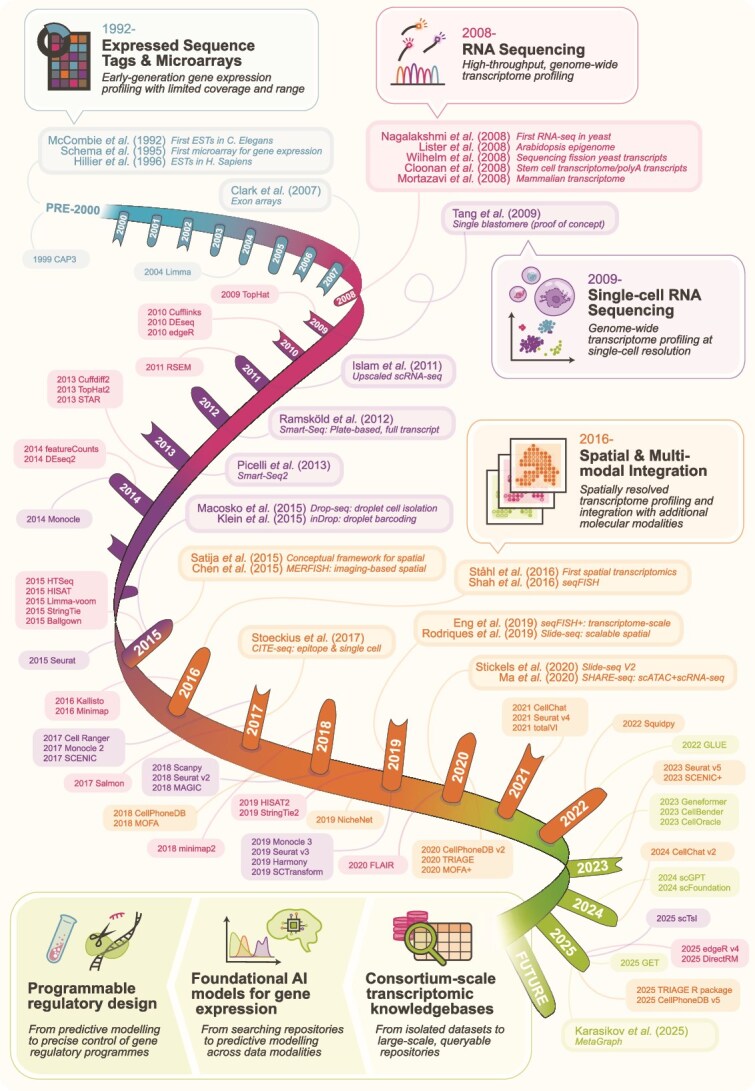
Overview of the past 25 years of gene expression analysis, including foundational milestones from the 1990s; four major technological transitions are shown: (i) expressed sequence tags and microarrays, (ii) RNA sequencing, (iii) single-cell RNA sequencing, and (iv) spatial transcriptomics and multimodal integration; landmark papers are indicated for each transition, and representative bioinformatics tools are listed along the timeline.

This Perspective summarizes how gene expression analysis has evolved by combining bibliometric trends with key technological milestones. We highlight how shifts in methodology, data scale, and analytical strategies have shaped gene expression analysis and set the stage for emerging computational paradigms that aim to transform how transcriptomic data are interpreted and used. Our discussion traces four key revolutions in this landscape: expressed sequence tags (ESTs) and microarrays, RNA sequencing (RNA-seq), single-cell transcriptomics, and spatial and multimodal integration (Fig. 1). We then examine the challenges and opportunities that characterize the present era and motivate the future directions discussed in this Perspective.

Looking forward, we argue that the next era of gene expression analysis will be defined less by the generation of new data and more by how we organize and reuse transcriptional and multimodal information. We outline three future directions: (i) consortium-scale searchable transcriptomic knowledgebases that bridge data generation and data reuse; (ii) foundation models for gene expression analysis that generalize across systems and species; and (iii) programmable regulatory design, in which analytic insights are used to engineer gene expression. The field is shifting from descriptive measurement towards queryable, predictive, and programmable gene expression biology.

## A historical trajectory of gene expression analysis

Over the past 25 years, gene expression analysis has been shaped by successive methodological breakthroughs that expanded the scale and resolution with which transcriptional activity can be measured. Four major technological transitions define this trajectory: (a) the ESTs and microarray era from the early 1990s to mid-2000s, (b) the advent of RNA-seq from 2008 onwards, (c) the development and rapid adoption of single-cell transcriptomic technologies (initially demonstrated in 2009 and scaled substantially thereafter), and (d) the emergence of spatial transcriptomics and multimodal integration (from 2015 onwards). These four transitions reshaped both experimental practice and the conceptual understanding of gene expression analysis, laid the foundation for the data-rich environment that now enables integrative analysis, large-scale knowledgebases, and emerging predictive modelling approaches.

Given the breadth of gene expression analysis over the past 25 years, the examples highlighted are intended as representative. We acknowledge that some important studies may not be discussed, and any omissions are unintentional.

### The early era: expressed sequence tags and microarrays

While reverse transcription polymerase chain reaction (RT-PCR) and later quantitative RT-PCR (qRT-PCR) enabled single-gene quantification, the origins of high-throughput gene expression profiling trace back to ESTs, which represented partial complementary DNA (cDNA) sequences corresponding to expressed genes. First reported in *Caenorhabditis elegans* [3] in 1992 and later extended to humans [1] in 1996, EST projects were the first systematic attempts to map gene activity across tissues and conditions. Although they could achieve high throughput, ESTs were inherently limited by only capturing short regions of transcripts and therefore could not distinguish between different isoforms from the same gene. These early efforts nevertheless established the foundations for gene discovery and annotation and directly contributed to the first genome assemblies and databases [16, 17].

The next leap forward came with the invention of microarrays. In 1995, this technology was introduced that allowed thousands of genes to be measured simultaneously on a glass chip using fluorescent hybridization [18]. Compared to ESTs, microarrays provided a more systematic and cost-effective way to survey genome-wide gene expression changes across conditions, revealing co-expressed gene clusters [19], tissue-specific signatures [20], and transcriptional responses to stimuli [21]. Despite their transformative impact, microarrays were limited by their reliance on predefined probes, which restricted discovery to known genes. Cross-hybridization artefacts also affected quantitative accuracy [22]. Later designs such as exon arrays [23] improved resolution but did not remove these fundamental constraints.

**Figure 2:**
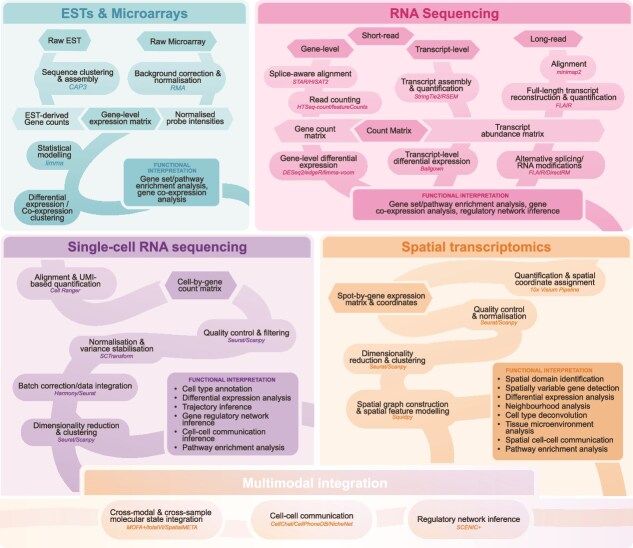
Gene expression analysis frameworks across four major transcriptomic technologies; typical computational workflows for analysing data generated from ESTs and microarrays, bulk RNA sequencing, single-cell RNA sequencing, and spatial transcriptomics are shown; representative tools used at different stages of the workflows are indicated.

This era also witnessed the development of early bioinformatic frameworks: EST analysis relied on sequence clustering and assembly tools such as CAP3 [24], while microarray data analysis was driven by statistical methods for differential expression, most notably the limma framework [25, 26], which became a cornerstone of microarray analysis (Fig. 2).

### The RNA sequencing revolution

A pivotal revolution occurred in 2008, when multiple independent studies simultaneously demonstrated that cDNA could be directly sequenced to generate quantitative maps of the transcriptome [27–31]. Unlike hybridization-based approaches, RNA-seq required no prior knowledge of gene annotations and provided nucleotide-level resolution, enabling the discovery of transcript structures and isoform diversity. Early applications across yeast [27, 28], *Arabidopsis thaliana* [29], and mammalian systems [30, 31] revealed a far greater transcriptional complexity than previously recognized, uncovering widespread alternative splicing and a rich diversity of previously unannotated noncoding RNAs.

The first wave of RNA-seq technologies was dominated by short-read sequencing, which rapidly increased in throughput. This expansion in data volume prompted the development of new analytical pipelines for alignment, quantification, and statistical testing. A wave of bioinformatics tools appeared, with representative examples including STAR [32], TopHat [33], TopHat2 [34], HISAT [35], and HISAT2 [36] for splice-aware alignment, HTSeq-count [37] and featureCounts [38] for read quantification, and DESeq [39], DESeq2 [40], and edgeR [41–44] for differential expression analysis (Fig. 2). For transcript-level quantification, RNA-Seq by Expectation-Maximization (RSEM) provided one of the earliest robust expectation-maximization frameworks for estimating transcript abundances with or without a reference genome [45]. A subsequent shift towards pseudo-alignment led to tools such as kallisto [46] and Salmon [47], which offer orders-of-magnitude faster performance for transcript quantification. In addition, end-to-end workflows such as the Tuxedo suite (TopHat-Cufflinks-Cuffdiff) [33, 48, 49] and its successor (HISAT-StringTie-Ballgown) [35, 50, 51] further streamlined the analysis of short-read RNA-seq data with improved speed, resource efficiency, and accuracy.

Despite these advances, the inherent read-length limitations of short-read RNA-seq restricted the reconstruction of full-length transcripts. This limitation stimulated the development of long-read sequencing technologies, which provided a complementary avenue for capturing complete transcript structures directly. Early platforms such as 454 generated reads several hundred base pairs in length, enabling initial attempts at full-length transcript reconstruction. Subsequent third-generation sequencing technologies, most notably PacBio single-molecule real-time sequencing [52] and Nanopore sequencing [53], provided reads extending to tens or even hundreds of kilobases. These technologies enabled the characterization of full-length transcripts [54] and, in the case of Nanopore, direct RNA-seq and the detection of RNA modifications [55]. This was accompanied by bioinformatics tools designed for long-read RNA-seq analysis, such as minimap [56] and minimap2 [57] for read alignment, StringTie2 [58] and FLAIR [59] for isoform reconstruction and quantification, and DirectRM [60] for modification detection (Fig. 2).

However, long-read sequencing technologies are associated with higher per-base error rates than short-read approaches. Although modern alignment algorithms are designed to tolerate sequencing errors [57], residual inaccuracies can complicate splice-site identification and the delineation of transcript boundaries, particularly for low-abundance transcripts. These uncertainties may affect isoform assignment and quantification accuracy. In the context of Nanopore direct RNA-seq, base-calling errors and signal noise can further complicate the reliable detection of RNA modifications. In practice, achieving robust isoform reconstruction and RNA modification detection often requires higher sequencing depth, hybrid strategies integrating short- and long-read data, or consensus-based error correction that increase both experimental cost and computational complexity.

RNA-seq transformed gene expression analysis from single gene amplicons and probe-limited surveys into comprehensive genome-wide measurements, enabling the systematic comparison of transcriptomes across conditions, tissues, and species. However, because bulk RNA-seq measures average expression across thousands of cells, it cannot resolve the underlying cellular heterogeneity, motivating the emergence of single-cell transcriptomic technologies.

### The rise of single-cell transcriptomics

The first demonstration of whole-transcriptome sequencing from a single cell was reported in 2009, showing that gene expression could be captured from picogram quantities of RNA and confirming the feasibility of cellular-resolution transcriptomics [5]. Although early implementations were limited to one cell per reaction, this proof-of-concept laid the foundation for scalable single-cell RNA sequencing (scRNA-seq). In the following years, the introduction of multiplexed library preparation protocols enabled the parallel processing of dozens to hundreds of cells using plate-based methods such as Smart-seq [61]. This then scaled to hundreds of thousands to millions of individual cells with droplet-based platforms, including Drop-seq [62], inDrop [63], and the 10x Genomics system [64], which use microfluidic droplets and cell-specific barcodes to enable large-scale parallel profiling. This advance expanded the ambition of biological studies, enabling analyses of rare cell populations [65], tissue heterogeneity [66], and large-scale perturbation screens [67, 68], among others.

While scRNA-seq captures rich expression profiles per cell, it requires efficient dissociation of intact live cells, a process that may selectively lose fragile cell types and is difficult to apply to samples where viable cells cannot be recovered. To address this limitation, single-nucleus RNA sequencing (snRNA-seq) was subsequently developed [69]. snRNA-seq offers a practical solution by enabling sequencing samples prone to distortion from cell dissociation including frozen tissues and postmortem samples, despite its own challenges such as nuclear biases, higher sparsity, and ambient RNA contamination [70, 71].

With the rise of single-cell transcriptomics, scRNA-seq datasets grew rapidly in scale and complexity, necessitating efficient computational solutions. To meet this need, a comprehensive ecosystem of computational tools has been developed, spanning raw data processing to downstream interpretation (Fig. 2). For example, Cell Ranger [64] provides raw data processing capabilities for 10x droplet-based platforms, including FASTQ generation and alignment to a reference genome. Seurat [72] and Scanpy [73] offer end-to-end workflows in R and Python, respectively, supporting quality-control, normalization, scaling, dimensionality reduction, cell clustering, integration, and more. Within these frameworks, SCTransform [74] has become a widely adopted method for modelling technical variation and stabilizing gene-wise variance. Dimensionality reduction algorithms such as t-distributed Stochastic Neighbour Embedding (t-SNE) [75] and Uniform Manifold Approximation and Projection (UMAP) [76] are also routinely incorporated into these workflows. In addition, trajectory-inference methods, such as the Monocle series [9, 77, 78], have become integral components of contemporary single-cell analysis pipelines.

Equally important is understanding the unique challenges associated with scRNA-seq and snRNA-seq, and how computational tools mitigate them. A major challenge in single-cell data is sparsity arising from expression dropouts, which fundamentally affects downstream analytical tasks. Inflated zero counts can distort gene–gene correlation structures, thereby influencing gene regulatory network inference. Sparsity may also obscure lowly expressed but biologically important regulators, leading to incomplete reconstruction of regulatory hierarchies. In trajectory inference, dropout may blur subtle transitional states and affect pseudotime prediction, particularly when dynamic genes are expressed at low levels. To address this, imputation and denoising approaches such as MAGIC [79], which uses diffusion-based smoothing to recover missing transcripts, and scTsI [80], a two-stage imputation method designed to improve gene–gene correlation structure, help alleviate zero inflation and improve downstream interpretability. Beyond dropout correction, Harmony [81] corrects batch effects to enable biologically meaningful integration across datasets, while CellBender [82] has become a widely adopted approach for removing ambient RNA contamination, particularly in snRNA-seq data.

Despite these challenges, the depth of biological insight enabled by the rise of single-cell transcriptomics is unmistakable. It has reshaped how we understand cellular heterogeneity, developmental trajectories, and disease mechanisms, revealing cell states, cell-specific regulatory programmes, and cell–cell interactions that were previously inaccessible. The impact of single-cell data on biological research has already been profound, and as technologies advance and datasets grow in scale, their influence is expected to expand even further.

### The era of spatial and multimodal integration

Single-cell approaches resolved cellular heterogeneity, but they often lost the spatial relationships that underpin tissue architecture. This motivated the development of spatial transcriptomics. Although earlier histological and *in situ* hybridization methods provided spatial information, the ability to quantify mRNA expression for large numbers of genes within intact tissues only emerged in the mid-2010s [83]. Imaging-based technologies such as seqFISH and MERFISH demonstrated that individual RNA molecules could be measured directly in their native cellular context with increasingly multiplexed capacity, providing a conceptual foundation for modern spatially resolved transcriptomics [84–86]. In 2015, the first computational framework for reconstructing spatial organization was introduced by integrating scRNA-seq profiles with *in situ* hybridization data [6]. In parallel with these advances, a distinct experimental strategy was reported in 2016, when Ståhl *et al*. introduced the first widely adopted barcoded array method for spatial transcriptomics [7]. This method provided the first practical platform for spatially resolved whole-transcriptome sequencing.

Recent years have seen rapid expansion of spatial transcriptomics platforms. Sequencing-based platforms such as 10x Genomics Visium systems, Slide-seq [87], and Slide-seqV2 [88] have enabled high-throughput, whole-transcriptome profiling across intact tissues. Meanwhile, imaging-based platforms such as seqFISH+ [89] achieve subcellular spatial resolution through highly multiplexed *in situ* hybridization strategies. These innovations have transformed spatial transcriptomics into a versatile framework for mapping gene expression within its native tissue context and have catalysed a wave of studies dissecting cellular neighbourhoods and spatially organized regulatory programmes.

Despite rapid technological progress, spatial transcriptomics platforms are constrained by trade-offs between spatial resolution, transcriptome coverage, and spatial throughput. Sequencing-based platforms typically capture transcripts from spots containing multiple cells, generating mixed-cell signals that complicate cell-type deconvolution. Conversely, imaging-based platforms achieve subcellular resolution but often rely on predefined gene panels, which constrain transcriptome breadth, and require iterative imaging cycles that can limit practical spatial throughput when scaling to large tissue areas. These trade-offs propagate into downstream analyses by introducing cell mixing, incomplete gene representation, and resolution-dependent sparsity, thereby influencing the accuracy of spatial domain detection, neighbourhood inference, and the identification of spatially variable genes.

A typical analysis workflow extends standard single-cell processing by modelling the spatial coordinates and tissue neighbourhood graph. Downstream tasks include the identification of spatial domains (regions with coherent transcriptional programmes), detection of spatially variable genes, and the characterization of microenvironments through local co-expression and neighbourhood composition. In practice, end-to-end workflows are available in both R and Python. Seurat supports preprocessing, integration, and spatial analyses within the Seurat object framework in R, whereas Scanpy [73] and Squidpy [90] build on the AnnData Python framework to provide spatial neighbour graphs, identify spatially variable genes, and perform neighbourhood enrichment or co-occurrence analyses of cell-type organization (Fig. 2).

While spatial transcriptomics integrates gene expression profiles with positional context, a broader landscape of multimodal technologies has emerged that combines transcriptomics with genetic variation, chromatin accessibility, epigenetic modifications, and proteomic or metabolic readouts. These approaches capture multiple layers of cellular regulation, developmental processes, and disease mechanisms, positioning gene expression as a central hub that links upstream regulatory mechanisms to downstream phenotypic states. This has driven the development of integrative computational frameworks capable of leveraging multimodal datasets. For example, the integration of genotype and expression data has reshaped how genetic variation is interpreted by enabling expression quantitative trait locus (eQTL) mapping and transcriptome-wide association studies that connect regulatory variants to downstream phenotypes [91]; single-cell transcriptomic data can be combined with receptor-ligand resources to infer cell–cell communication [92–94]; gene expression profiles can be integrated with histone modification maps to identify context-specific regulatory genes [95]; and joint analysis of scRNA-seq with single-cell chromatin accessibility can be used to infer enhancer activity and gene regulatory networks [10]. In addition, broader factor-based models such as MOFA [96] and MOFA+ [97], as well as probabilistic frameworks such as totalVI [98] for the joint modelling of transcriptomic and proteomic measurements, highlight the growing toolbox for interpreting gene expression in a broader molecular context.

## Bibliometric insights

**Figure 3:**
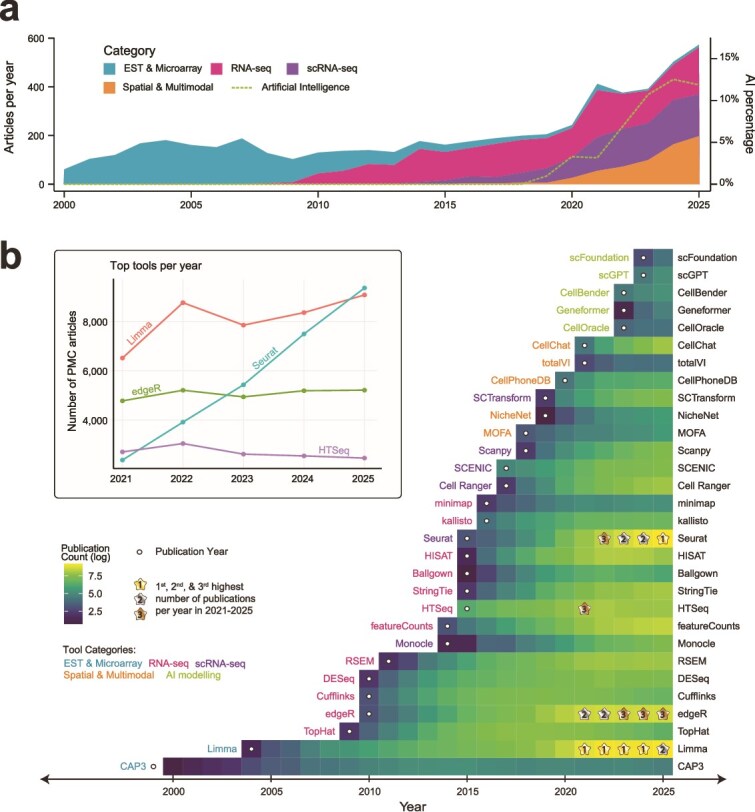
Bibliometric trends over the past 25 years of gene expression analysis; (a) publication trends for gene expression analysis from 2000 to 2025 in six leading bioinformatics journals: ‘Briefings in Bioinformatics’, ‘Bioinformatics’, ‘Nature Biotechnology’, ‘Genome Biology’, ‘Genome Research’, and ‘Nucleic Acids Research’; the number of articles in each category is shown over time (left *y*-axis), and the proportion of articles involving AI-based methods is shown as a green dashed line (right *y*-axis); two key surges in AI usage and relevant developments driving them are annotated in green; (b) annual number of PubMed Central open-access full-text articles that mention each tool from 2000 to 2025; white circles indicate the tool’s publication year; although some tools were publicly available prior to formal publication, counts are shown only from the publication year onwards to enable more consistent comparisons across tools; the top three tools in each of the most recent 5 years are highlighted, and their usage trends are shown in the accompanying line plot (inset).

Over the past 25 years, the bibliometric landscape of gene expression analysis has closely tracked the methodological transitions outlined above. A survey of publications in six leading bioinformatics journals, which are strong indicators of methods development, delineates four broad phases: an early-2000s period dominated by microarray- and EST-based expression profiling; a subsequent rise of bulk RNA-seq beginning around 2008 that progressively displaced microarrays and ESTs; and, after 2010, the emergence of scRNA-seq, as well as spatial profiling and multimodal integration, which together with RNA-seq now constitute three major, co-existing paradigms in the field of gene expression analysis (Fig. 3a).

Notably, the involvement of artificial intelligence (AI) has increased steadily since 2018, with two surges observed in 2018–20 and 2021–24 (Fig. 3a). The first surge coincides with the introduction of the transformer architecture in 2017 [99], aptly titled ‘Attention Is All You Need’, which accelerated the development of large-scale pretraining and foundation-model approaches. The second surge is likely attributable to the widespread visibility and adoption of large language models, amplified by the release of GPT-3 in 2020 and the launch of consumer-facing chatbots such as ChatGPT in 2022, which drew greater attention within the research community to leverage the power of AI in biological research.

To place tools on the same timeline, we analysed 70 78 831 open-access full-text articles in PubMed Central and counted the number of articles in which each tool was mentioned each year from 2000 to 2025. This metric reflects the popularity of bioinformatics tools within the research community and more directly captures the application side. Limma drew the most sustained attention over the past two decades and ranked as the most popular tool overall. In contrast, Seurat rose sharply in recent years and overtook limma in 2025 (Fig. 3b). In recent 5 years, the three most frequently mentioned tools were limma, Seurat, and edgeR (Fig. 3b). This pattern may seem unexpected, but it is consistent with the broad reach of limma’s linear modelling framework. Limma was originally developed for microarrays, but it generalizes across a wide range of experimental designs in later developments. Extensions such as limma-voom make it well suited to RNA-seq, supporting robust differential expression analyses even with limited replication by borrowing information across genes [25]. Meanwhile, the growing prominence of scRNA-seq and spatial transcriptomics has favoured end-to-end analysis ecosystems, and Seurat’s continued adoption reflects sustained development by the Satija Lab across multiple major releases, from Seurat v1 (2015) to Seurat v5 (2023-present), which expands support for scalable single-cell analysis and for spatial and multimodal workflows [100]. Finally, the enduring popularity of edgeR aligns with its mature and actively maintained statistical framework for count-based differential analysis using negative binomial models and generalized linear models, which remains widely used for RNA-seq and other sequencing-based assays [44]. In parallel, HTSeq, particularly the htseq-count module, has long provided a reliable Python-based framework for read counting as an upstream step feeding into these statistical models [37].

## Current challenges

While these technological advances have dramatically expanded the scope of gene expression analysis, they have also created new challenges for ensuring robust, reproducible, and scalable analytical frameworks. New methods for gene expression analysis are reported at a rapid pace, yet comparative evaluations are often narrow in scope, and relatively few tools mature into broadly adopted community standards. Many bioinformatics tools struggle to translate technical novelty into day-to-day usability, and are short-lived and receive limited maintenance after publication, underscoring the need for sustained support and a healthier software ecosystem. Leading bioinformatics journals could play a more active role by encouraging usable software, long-term maintenance, robust documentation, and stable releases, as illustrated by widely adopted tools such as edgeR and Seurat.

Even when widely used toolkits exist, default parameter choices may not transfer across datasets. For example, Seurat is a widely used R package for scRNA-seq data analysis, yet parameter choices such as the number of principal components, the clustering algorithm, and the clustering resolution can yield different outcomes across datasets and often require dataset-specific tuning. However, practical guidance on how to tune these parameters and which diagnostics to prioritize is often limited, and as a result many users, particularly less experienced ones, rely on default settings. Reproducibility and transparency also remain uneven. Although many tools are open-source, full reproducibility of published analyses often relies on complex software environments, undocumented parameter settings, or access to intermediate processing steps. The increasing use of black-box AI models further complicates interpretability and trust.

Addressing this challenge will likely require clear, enforceable standards in model design, and tool usage, particularly with respect to practices such as parameter reporting, evaluation frameworks, and reproducible analysis workflows. At a minimum, analyses should report preprocessing steps, software versions, and key parameter choices in a transparent and reproducible manner. Containerized workflows can improve computational reproducibility, while community-maintained benchmark datasets and standardized evaluation metrics can facilitate fair comparison across methods. Broader discussions in computational biology have emphasized the importance of transparent parameter reporting, code availability, and workflow documentation [101, 102]. Journals and funding agencies may further strengthen these practices by requiring code deposition, reproducibility checklists, and benchmarking against shared reference datasets. For example, journals in the Nature portfolio have introduced reporting standards requiring the availability of data, materials, code, and protocols to support transparency and reproducibility.

Progress towards improved robustness and standardization is also emerging in specific subfields. In scRNA-seq analysis, consensus clustering strategies, such as SC3 [103], have been proposed to mitigate sensitivity to parameter choices and improve clustering stability. Similarly, curated expression portals that apply consistent quality-control and processing pipelines before downstream modelling illustrate the importance of standardized data governance [104]. These developments suggest that although progress towards improved reproducibility and standardization is emerging, such efforts remain fragmented and often subfield-specific rather than representing universally adopted standards across computational genomics.

Lastly, as high-throughput transcriptomic data generation becomes increasingly routine, a major rate-limiting step is shifting towards experimental validation to establish causation rather than association. Predictive models for perturbation response, including recent foundation-model approaches (e.g. GeneFormer [105]), can help de-risk this process by prioritizing candidate interventions and expected expression changes, but functional validation remains essential. Closing this gap will require tighter computational and experimental loops as well as shared benchmarks that link perturbational outcomes to transcriptomic readouts. In parallel, distinguishing high-quality from low-quality datasets remains challenging as public resources expand. Clear, consistently applied quality-control criteria and, where feasible, biological and technical replicates are essential to ensure downstream inferences reflect biological signal rather than technical artefacts.

## Future outlook

Rapid progress in transcriptomic technologies and analytical frameworks has not only transformed how gene expression is measured and interpreted but is also opening new frontiers. Here, we highlight three emerging directions that we anticipate will be particularly influential.

### Consortium-scale searchable transcriptomic knowledgebases

The volume of high-throughput sequencing data stored in public repositories has reached petabase scale, spanning DNA, RNA, and protein sequences, as well as other molecular modalities across all major branches of life. Yet most of these data remain ‘dark’, as they are archived but not readily searchable at the resolution needed for integrative analysis. Traditional workflows still treat new experiments as self-contained, with limited systematic reuse of the vast corpus of existing data. A major driver of this transition is the rise of large consortium-scale efforts that generate standardized datasets intended for broad reuse rather than single-study conclusions. Flagship initiatives such as the Human Cell Atlas aiming to build comprehensive reference maps of human cells while establishing shared conventions for sampling, metadata, and annotation [106]. In parallel, population-scale resources such as the Genotype-Tissue Expression project provide systematic, multi-tissue genotype-expression references for eQTL mapping and for interpreting genome-wide association studies (GWAS) signals by prioritizing candidate causal genes [107]. These consortium-scale resources provide curated, standardized substrates for building the searchable transcriptomic knowledgebases envisioned here.

Recent advances in petabase-scale sequence search, such as frameworks based on annotated de Bruijn graphs and compressed full-text indexes, demonstrate that it is now technically feasible to index tens of millions of sequence datasets and perform full-text search across tens of petabase pairs of raw sequence data [108]. These developments suggest that the technical barriers to large-scale sequence search are no longer the primary bottleneck, making it increasingly feasible to couple data generation with systematic data search and reuse as a system rather than as separate stages.

The next decade is likely to see the emergence of consortium-scale, and ultimately global-scale, searchable transcriptomic knowledgebases that unify multimodal data across tissues, species, and perturbations. For gene expression analysis, such infrastructures could enable scalable and queryable transcriptomic knowledgebases. Instead of asking whether a gene is differentially expressed in a single study, researchers could conveniently query across thousands of studies to identify when, where, and under what conditions similar expression patterns occur. Cell-type-specific signatures, rare cell states, or regulatory modules could be identified by searching embedding spaces derived from unified expression profiles. This shifts gene expression analysis from study-centric workflows to query-centric reuse across repositories. New datasets will be able to plug into the existing transcriptomic knowledgebases and be embedded into a shared latent space that can be easily queried. Such infrastructures will be essential for realizing the potential of foundation models and for ensuring that future analytical advances translate into reusable, community-wide resources.

### Foundation models for gene expression analysis

AI has expanded rapidly in recent years and started to redefine gene expression analysis. Deep learning architectures, such as deep generative models, transformer-based models, and graph neural networks, are now capable of multimodal data integration, capturing non-linear dependencies in gene regulatory networks, predicting perturbation responses, and enabling cross-species transfer learning in gene expression analysis, among other applications [105, 109–111]. These models provide a powerful computational framework for modelling gene expression landscapes. However, existing models are trained for specific tasks on limited datasets and therefore do not yet function as broadly applicable foundation models for gene expression analysis.

Recent benchmarks in gene perturbation effect prediction suggest that deep-learning-based foundation models do not consistently outperform simple linear baselines [112]. Importantly, this finding is task-specific and should not be generalized to all applications of deep learning in gene expression analysis. Different tasks, such as cross-dataset integration and multimodal integration, may involve complex non-linear structure that is difficult to capture using linear models alone. Nevertheless, these results underscore the need for rigorous evaluation frameworks, including diverse benchmarking datasets and transparent reporting of performance across metrics that reflect biological utility.

More broadly, the practical value of foundation models in gene expression analysis will depend on addressing several key challenges. First, training data bias remains a central concern. Large-scale transcriptomic datasets are often skewed towards specific tissues, species, disease contexts, or experimental platforms, which may limit generalizability across biological systems. Addressing this issue will require careful dataset curation, consistent preprocessing, and metadata standardization, as well as clear reporting of training data composition to ensure representativeness across experimental contexts. Second, uncertainty quantification will be important for practical decision-making. Predictions from foundation models should be accompanied by calibrated confidence estimates. Emerging approaches, including Bayesian approximation, ensemble modelling, and probabilistic prediction frameworks [113], offer potential strategies for uncertainty quantification, although well-calibrated uncertainty estimation in large-scale foundation models remains an active area of research. Third, biological interpretability will be critical for experimental adoption. Rather than acting as opaque oracles, foundation models should provide biologically meaningful decompositions by highlighting key evidence, such as regulons and pathways, that drive the predictions, and should integrate seamlessly with experimental design, guiding which conditions or readouts are most informative to measure next. In this way, foundation models can transform gene expression analysis from a descriptive activity into a predictive engine that informs experimental strategy.

In parallel, questions surrounding the long-term sustainability of pretrained model repositories are likely to become increasingly relevant, as AI models continue to develop and see broader adoption. While many models are currently distributed through commercial code-hosting platforms or project-specific websites, relatively few are maintained within long-term, publicly supported infrastructures. The discontinuation of community-driven platforms such as Kipoi [114] underscores the vulnerability of model-sharing infrastructures that lack sustained institutional support. As reliance on large-scale pretrained models grows, co-ordinated public investment, including support from national funding agencies such as the National Institutes of Health (NIH) and comparable international bodies, may help establish durable repositories. Such repositories would support reproducibility, benchmarking, and long-term translational impact.

Looking ahead, we anticipate the emergence of foundation models for gene expression analysis, analogous to large language models in natural language processing. Consortium-scale transcriptomic knowledgebases, integrating data across tissues, species, perturbations, and experimental platforms, are likely to form a critical substrate for training such models. By learning generalizable representations of gene regulatory states from such data, these models could extend beyond task-specific optimization and support zero-shot annotation of new datasets, cross-species mapping of cell types, and the prediction of responses to unseen combinations of perturbations. With continued advances in data standardization, uncertainty quantification, and biological interpretability, foundation models may ultimately offer a unifying computational framework for modelling gene expression across biological systems, bridging descriptive atlases with predictive and experimentally actionable insights.

### From analysis-to-control: programmable regulatory design informed by gene expression

Advances in gene expression analysis enable the rational engineering of gene regulatory programmes. This shift is occurring alongside rapid progress in synthetic biology and nucleic-acid therapeutics, which together are expanding the space of controllable interventions from DNA-level regulatory element engineering and gene therapy to RNA medicines that directly modulate transcripts. For example, cell-type-selective regulatory DNA elements can be used to constrain transgene expression to specific lineages or states, improving both efficacy and safety in gene delivery settings [115–118]. Among RNA-targeting modalities, ASOs offer a clinically attractive route because their effects can often be titrated and are, in many contexts, reversible [14]. Collectively, they support a view in which gene expression becomes an engineerable variable in therapy, and programmable regulatory design provides the common language to connect molecular modalities with clinical objectives.

As transcriptomic knowledgebases and predictive models mature, gene expression analysis will be positioned to inform how cellular states can be engineered. This ‘analysis-to-control’ transition is driven by two converging trends. First, large-scale bulk, single-cell, and spatial resources provide increasingly precise maps of where genes are expressed and which regulatory programmes specify cell identity. Second, AI-enabled models and integrative multimodal frameworks are beginning to translate these observational patterns into actionable hypotheses about which perturbations are most likely to shift a cell from one transcriptional state to another in a predictable manner. These developments make it feasible to define control objectives for gene expression and to design interventions that are tailored to specific cellular contexts. In practice, transcriptomic state descriptions can be reframed as engineering specifications, in which desired expression levels, cell-type selectivity, and safety constraints are treated as design criteria rather than post hoc readouts. Successful deployment will depend on interpretability and causal grounding. The field will benefit from design objectives that incorporate uncertainty, off-target risk, and biological feasibility constraints, such as chromatin accessibility and cell-state-dependent regulatory logic.

Overall, transcriptomics contributes value by enabling target selection, context-specific prioritization, and rational design of interventions across DNA-level regulatory element engineering, gene therapy, and RNA medicine. Nevertheless, a major rate-limiting step remains functional validation. While transcriptomic knowledgebases and predictive models can help prioritize perturbations and reduce the risk of experimental failure, establishing causation still requires rigorous wet-lab confirmation. Validated outcomes can then be used to refine models and improve predictive performance over time. Bridging analysis and control will therefore require tightly coupled computational-experimental loops, standardized benchmarks, and shared resources that connect expression phenotypes to perturbational outcomes.

## Methods

### Bibliometric analysis

We queried PubMed for articles published between 2000 and 2025 in six leading journals in bioinformatics: ‘Briefings in Bioinformatics’, ‘Bioinformatics’, ‘Nature Biotechnology’, ‘Genome Biology’, ‘Genome Research’, and ‘Nucleic Acids Research’. To ensure consistent temporal coverage and comparability across journals over the entire study window (2000–25), we restricted this publication-trend analysis to journals that were continuously indexed throughout the period. Therefore, ‘Nature Methods’ and ‘PLOS Computational Biology’ were not included because they launched after 2000. We acknowledge that excluding later-launched journals may omit some methodological innovations. Nevertheless, a sensitivity analysis including these journals produced similar overall trends. We selected leading bioinformatics journals as they are strong indicators of methods development and therefore offer a useful lens for tracking methodological advances in gene expression analysis. To map technological transitions over time, we performed keyword searches in titles and abstracts (field tag: [tiab]) and assigned articles to four categories: (1) ESTs and microarrays, (2) bulk RNA-seq, (3) scRNA-seq, and (4) spatial transcriptomics and multimodal integration. Full Boolean query strings for each category (including all synonyms and spelling variants) are provided in the accompanying source code. Since the RNA-seq query may capture some scRNA-seq papers (owing to overlapping terminology), we removed records from the RNA-seq set that matched only the scRNA-seq keyword pattern and did not match any bulk RNA-seq specific terms. To estimate the involvement of AI in each category, we screened titles and abstracts using a set of keywords capturing common AI terminology (e.g. ‘graph neural network’ and ‘transformer’, full patterns are listed in the source code). For each year, we computed (i) the number of publications in each category and (ii) the proportion of publications matching at least one AI pattern. These summaries were used to generate Fig. 3a using custom R scripts.

To characterize trends in tool visibility over time, we analysed 70 78 831 open-access full-text articles in PubMed Central published between 2000 and 2025. For each tool, we used the tool name as a keyword and counted the number of articles mentioning it each year. This corpus-wide analysis included all journals indexed in PubMed Central. We acknowledge that mention frequency was used as a proxy for longitudinal visibility in the literature rather than as a direct measure of actual usage or citation impact, and may therefore overestimate adoption for certain tools. For tools with multiple versions, we consolidated number of articles under the original tool name, e.g. HISAT2 and HISAT were both assigned to HISAT. To reduce false positives from ambiguous or generic terms, we excluded tools whose names frequently matched unrelated contexts in full text, including STAR, TRIAGE, Salmon, GLUE, MAGIC, Harmony, and FLAIR. We also excluded tools first published in 2025 to avoid bias from partial-year coverage. This survey was designed to track a curated set of representative tools in gene expression analysis spanning the four technological transitions, rather than to exhaustively enumerate all bioinformatics tools. The resulting annual counts were used to generate Fig. 3b.

## Data Availability

The analysis workflow, source code, intermediate files, and final output files generated during the bibliometric analysis are publicly available on GitHub: https://github.com/Qiongyi/Bibliometric_analysis.
